# Body size and brain volumetry in the rat following prolonged morphine administration in infancy and adulthood

**DOI:** 10.3389/fpain.2023.962783

**Published:** 2023-02-27

**Authors:** Milo Taylor, Anya Brooke Cheng, Duncan Jack Hodkinson, Onur Afacan, David Zurakowski, Dusica Bajic

**Affiliations:** ^1^ Department of Anesthesiology, Critical Care and Pain Medicine, Boston Children’s Hospital, Boston, MA, United States; ^2^ Harvard College, Massachusetts Hall, Cambridge, MA, United States; ^3^ Division of Clinical Neuroscience, School of Medicine, University of Nottingham, Nottingham, United Kingdom; ^4^ Sir Peter Mansfield Imaging Centre, School of Medicine, University of Nottingham, Nottingham, United Kingdom; ^5^ National Institute for Health Research (NIHR), Nottingham Biomedical Research Center, Queens Medical Center, Nottingham, United Kingdom; ^6^ Versus Arthritis Pain Centre, University of Nottingham, Nottingham, United Kingdom; ^7^ Department of Radiology, Boston Children’s Hospital, Boston, MA, United States; ^8^ Harvard Medical School, Boston, MA, United States

**Keywords:** infant, magnetic resonace imaging (MRI), segmentation, neonatal, brain volume, opioid

## Abstract

**Background:**

Prolonged morphine treatment in infancy is associated with a high incidence of opioid tolerance and dependence, but our knowledge of the long-term consequences of this treatment is sparse. Using a rodent model, we examined the (1) short- and (2) long-term effects of prolonged morphine administration in infancy on body weight and brain volume, and (3) we evaluated if subsequent dosing in adulthood poses an increased brain vulnerability.

**Methods:**

Newborn rats received subcutaneous injections of either morphine or equal volume of saline twice daily for the first two weeks of life. In adulthood, animals received an additional two weeks of saline or morphine injections before undergoing structural brain MRI. After completion of treatment, structural T2-weigthed MRI images were acquired on a 7 T preclinical scanner (Bruker) using a RARE FSE sequence. Total and regional brain volumes were manually extracted from the MRI images using ITK-SNAP (v.3.6). Regions of interest included the brainstem, the cerebellum, as well as the forebrain and its components: the cerebral cortex, hippocampus, and deep gray matter (including basal ganglia, thalamus, hypothalamus, ventral tegmental area). Absolute (cm^3^) and normalized (as % total brain volume) values were compared using a one-way ANOVA with Tukey HSD post-hoc test.

**Results:**

Prolonged morphine administration in infancy was associated with lower body weight and globally smaller brain volumes, which was not different between the sexes. In adulthood, females had lower body weights than males, but no difference was observed in brain volumes between treatment groups. Our results are suggestive of no long-term effect of prolonged morphine treatment in infancy with respect to body weight and brain size in either sex. Interestingly, prolonged morphine administration in adulthood was associated with smaller brain volumes that differed by sex only in case of previous exposure to morphine in infancy. Specifically, we report significantly smaller total brain volume of female rats on account of decreased volumes of forebrain and cortex.

**Conclusions:**

Our study provides insight into the short- and long-term consequences of prolonged morphine administration in an infant rat model and suggests brain vulnerability to subsequent exposure in adulthood that might differ with sex.

## 1. Introduction

There is a plethora of evidence linking untreated pain to both physical (1, 2) and psychological symptoms (3, 4) that are detrimental to neonates. Opioids such as morphine have been shown to both relieve acute pain in infants and reduce procedural complications (5, 6) and have become the “gold standard” for pain treatment in pediatric procedural and perioperative settings (7). In select cases, critically ill neonates and children receive prolonged opioid treatment as part of sedation, which facilitates ventilation and reduces anxiety, agitation, and stress (7–9). Although treatment of acute pain is the standard of care, our understanding of the effects of *prolonged* opioid administration in the developing brain on possible long-term sequelae is limited. Results from the Neurological Outcomes and Preemptive Analgesia in Neonate (NEOPAIN) trial (10) strongly suggest long-lasting adverse effects on body weight and head circumference, as shown in children ages 5–7 who were born prematurely and required intubation in the first 72 h of life (11). In addition, morphine-treated children had more social problems and exhibited increased response latency during a short-term memory test. A larger study by De Graaf et al. (12) that followed children up to 8–9 years of age did not observe morphine–associated adverse effects reported by the NEOPAIN trial (lower body weight and head circumference, increased social problems, and poorer executive functions). Since morphine administration rates were lower in the study by De Graaf et al. (12), it is possible that long-term sequelae related to morphine therapy are dose dependent. These results speak to a lack of consensus on the long-term effects of opioid dosing in infancy on behavioral and physiological markers.

Animal models provide a translational tool to explore the neurobiological effects of morphine treatment with greater control over experimental conditions. Typically, chronic morphine administration in human infants is associated with numerous comorbidities, including but not limited to prematurity and surgical interventions. In the absence of pain or comorbidities, rodents exposed to prolonged morphine administration for 7 days in infancy demonstrated increased nociceptive response to thermal and chemical stimuli in adulthood (13, 14). Similarly, our previous report showed lower thermal pain thresholds in adult rats following early prolonged morphine administration in the first two weeks of life (15) with no differences in mechanical thresholds. These findings implicate alterations in pain processing resulting from prolonged morphine treatment in infancy. In adult rats previously treated with morphine in infancy, subsequent morphine exposure has been shown to increase the analgesic effect (16), which suggests a modified response to opioid medications following chronic exposure in infancy. Many studies demonstrate not only the chemical but structural consequences of chronic morphine use in infancy, including decreased hippocampal cell division (17) and increased neuronal and microglial apoptosis (18). However, evidence of intact whole-brain *in vivo* structural changes that occur as a result of prolonged morphine administration in infancy are limited.

In this study using a rat model, we probe the morphological consequences of prolonged morphine administration in infancy on brain volumes throughout life. For rodent development, it is reported that the proliferation and migration of cells in rats begin at gestational day 9.5 and end at about postnatal day (PD)15 (19). Both the maturation and function of pain pathways, as well as the mechanisms of prolonged opioid effects in a rat model are age dependent. Specifically, increased excitability of nociceptive circuits peaks at postnatal day PD6 and decreases to an adult-like level by PD21 (20, 21). Furthermore, some of the mechanisms of opioid tolerance (22) and dependence (23, 24) partially correspond to those of adult rats at PD14 and are equivalent to an adult at the PD21. Therefore, we decided to expose developing rat pups to prolonged postnatal morphine administration during this early period of brain development (PD1–14) when mechanisms of pain perception and opioid treatment differ to those in adult rats. We hypothesized that infant rats treated with prolonged morphine would exhibit smaller total and regional brain volumes. We also hypothesized that these differences would resolve in adulthood and that subsequent morphine administration will not lead to brain size (mal)adaptation. Our objective was to evaluate the (1) short- and (2) long-term effects of prolonged morphine administration in infant rat on body weight and brain volume, and (3) to determine if prolonged morphine exposure in infancy leads to an increased vulnerability to morphine related effects in adulthood.

## 2. Materials and methods

### 2.1. Animal care and use

The Institutional Animal Care and Use Committee at Boston Children's Hospital approved the experimental protocols for the use of vertebrate animals in this study. Experiments were conducted according to the U.S. Department of Health and Human Services “Public Health Service Policy on Humane Care and Use of Laboratory Animals” (NIH Publication No. 15-8013, revised 2015) prepared by the National Institute of Health Office of Laboratory Animal Welfare. Our group has previously described the animal care protocol (15, 25, 26). Briefly, pregnant dams were received on day 18 and handled daily. Cages were checked at 9AM and 5PM daily and pups found at either time were deemed 0 days of age. This study used eight litters, each of which had between 9 and 12 pups. Rat pups were randomly assigned to pharmacological groups in a split-litter (within-litter) design with balanced pharmacological treatment distribution per litter (27, 28). Pups of both sexes were included in the study. Animals were housed with their litters and kept on a 12-hour light/dark cycle. Food and water were given *ad libitum*. One set of animals was analyzed in infancy, and a second set of animals was housed until adulthood. The latter group was weaned from dams at 3 weeks of age [postnatal day (PD)18] and sex-separated with 3–4 animals per cage.

### 2.2. Animal treatment

#### 2.2.1. Pharmacological treatment

The method used in this study to model prolonged morphine administration and associated morphine dependence (29) and analgesic tolerance (22) was originally described by Barr's group. The increased analgesic tolerance was subsequently confirmed in our lab (25). Morphine sulfate (10 mg/kg; Baxter Health Care Corporation, Deerfield, IL) or an equal volume of saline was administered subcutaneously in the upper or lower back. Our group used subcutaneous injections rather than intraperitoneal injections to minimize nociceptive experience from drug administration. We also extended the period of administration from 6½ (1 week) to 13½ days (2 weeks) (15). The first injection occurred on PD1, and animals received twice-daily injections (at 9 AM and 5 PM) from PD1 to PD14 (14 days). In adulthood, rats within each treatment group were assigned to a secondary pharmacological treatment group and received subcutaneous injections of either morphine or saline from PD56 to PD70. Injections were performed with either a 10- or 100- μl syringe (Hamilton Company, Reno, NV).

#### 2.2.2. Morphine weaning

**Figure 1:**
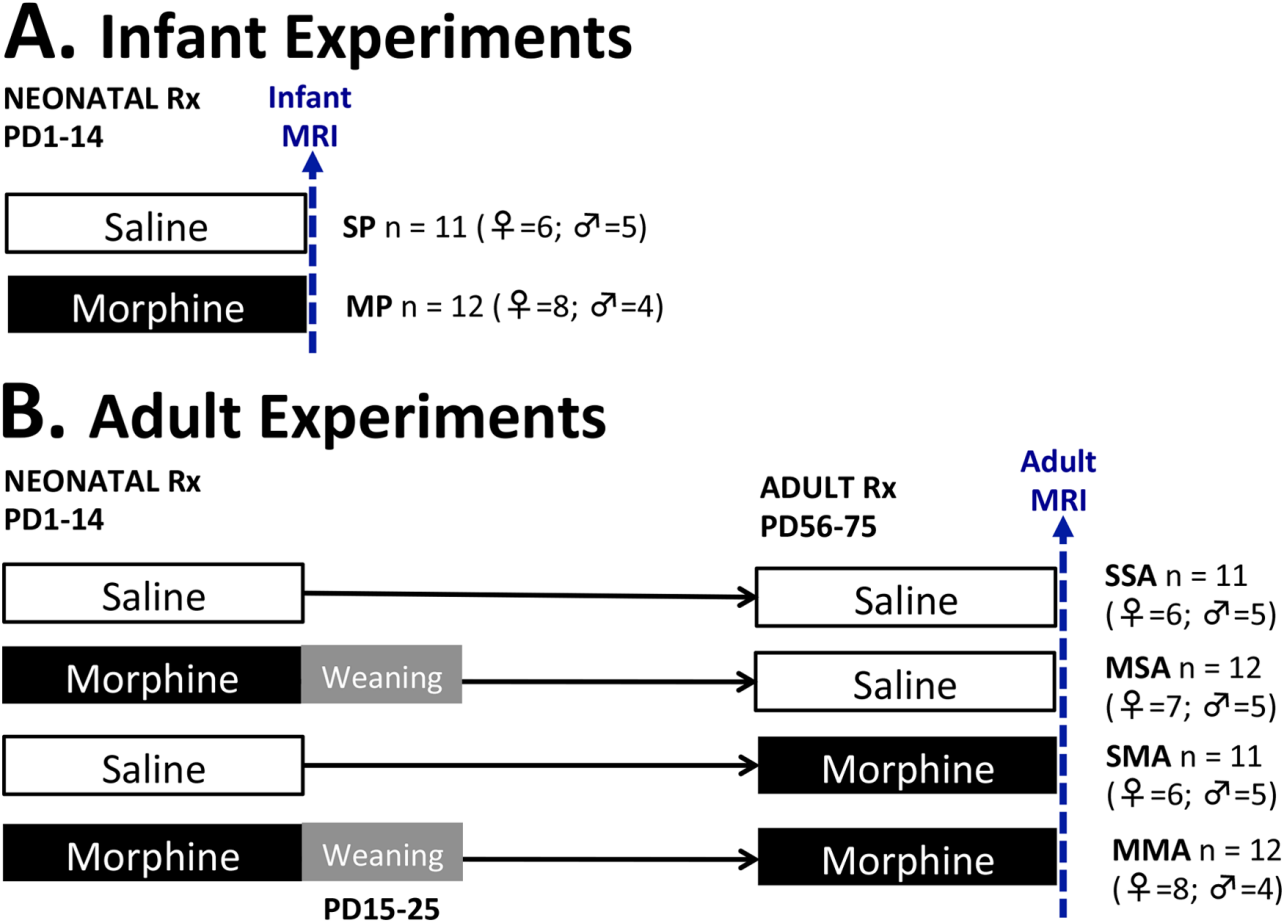
**Pharmacological groups.** Infant rat pups received treatment (Rx) with either morphine [morphine pups (MP); 10 mg/kg subcutaneously twice daily] or equivalent volume of saline [saline pups (SP)] starting on postnatal day (PD)1 for 2 weeks. One set of animals underwent brain MRI scan under light anesthesia on PD14 (**A**). Morphine-treated infant rats from another set underwent a 10-day weaning period from PD15-PD25 (gray area) and were allowed to growth to young adulthood when they received additional two weeks of treatment from PD56-PD75. The four adult groups were scanned after PD 57 (**B**) and included rats treated with saline in infancy and saline in adulthood (SSA); morphine in infancy and saline in adulthood (MSA); saline in infancy and morphine in adulthood (SMA); and morphine in infancy and adulthood (MMA).

Opioid dependence alters many physiological mechanisms, and abrupt discontinuation of morphine dosing results in withdrawal symptoms (30, 31). After the period of pharmacological treatment in infancy (PD 1–14), rats injected with morphine underwent morphine weaning for 10 days (PD15–24) to reduce potential withdrawal symptoms as described by Craig (15). Pups received incrementally decreasing morphine dosages: 3 days of twice-daily 5 mg/kg, 3 days of twice-daily 2.5 mg/kg, 2 days of twice daily 1.25 mg/kg, and 2 days of once daily 1.25 mg/kg. Signs of withdrawal were monitored using a scoring rubric developed by Gellert and Holtzman (32). No withdrawal symptoms were observed as a result of this protocol [Figure 1 in (15)].

#### 2.2.3. Pharmacological groups

The infant rats were divided into two treatment groups: saline-treated pups (**SP**, *n* = 6 females, *n* = 5 males) and morphine-treated pups (**MP**, *n* = 8 females, *n* = 4 males). The adult rats were divided into four treatment groups. The first adult group received injections of saline in infancy and adulthood (**SSA**, *n* = 6 females, *n* = 5 males). The next received morphine injections in infancy and saline injections in adulthood (**MSA**, *n* = 7 females, *n* = 5 males). Third group was treated with saline in infancy and morphine in adulthood (**SMA**, *n* = 6 females, *n* = 5 males), while the last group received morphine injection both in infancy and adulthood (**MMA**, *n* = 8 females, *n* = 4 males). The schematic Figure 1 illustrates the treatment groups. The immediate impact of pharmacological treatment differences was evaluated by performing an end-point analysis of infant groups (SP vs. MP). Long-term effects of infant morphine administration were explored in adulthood by analyzing the SSA and MSA groups. Lastly, by comparing the SSA, SMA, and MMA groups, we explored the effect of prolonged morphine administration in adulthood on total and regional brain volumes in the context of previous morphine administration in infancy.

### 2.3. Rodent magnetic resonance imaging (MRI)

#### 2.3.1. Anesthesia management

Infant rats were each anesthetized and scanned once between PD14 and PD17. Adult rats were scanned between PD69 and PD73. As previously described in detail (33), rats were anesthetized to minimize stress during scanning and reduce motion-related imaging artifacts. Rats were anesthetized with 3% Isoflurane/O_2_ at 1 L/min (Baxter Healthcare Corp., Deerfield, IL) prior to transportation to the MRI scanner. Once in the scanner, animals were placed on a warmed animal cradle (from a water bath heater at 50.4°C) in a prone position and reconnected to the anesthesia delivery system through a nose cone. The head was secured into a head restrainer with a built-in coil. A respiratory rate monitor was placed on the ventral chest and secured with paper tape. The whole system was advanced into the magnet. Once in the scanner, anesthesia was decreased and maintained at the lower level, (<1% Isoflurane/O_2_ at 1 L/min). Administration of O_2_ *via* a nasal cone at 1 L/min provided an estimated 24% fraction of inspired oxygen (FiO_2_). During imaging sessions, the level of anesthesia was gauged based on the respiratory rate using the *Small Animal Monitoring and Gating System* (Model 1025-S-50; Instruments Inc., San Diego, CA). The level of Isoflurane was titrated to a respiratory rate between 45 and 50 breaths per minute. Following completion of the scan, Isoflurane was discontinued, and animals were placed on a warming pad to recover (*Hot Dog Patient Warmer*; Augustine Biomedical and Design, Eden Prairie, MN).

#### 2.3.2. Brain MRI

Animals were scanned with a Bruker BioSpec 70/30USR 7 T MRI scanner (Bruker, Billerica, MA) at the *Small Animal Imaging Laboratory* at Boston Children's Hospital. For both infant and adult rats, we used a Bruker transmit-only volume coil with an inner diameter of 85 mm in combination with a 4-channel phased array receive-only surface rat brain coil (10–20 mm internal diameter; Bruker, Billerica, MA). We also used an anatomically shaped mouse brain array for infant rats, which in their third week are similarly sized to adult mice. T2-weighted structural images were acquired using a RARE (Rapid Acquisition with Relaxation Enhancement) FSE sequence [TE = 35 msec, TR = 4,000, FA = 90 degrees, RARE factor 8, FOV = 20 × 20 mm, matrix = 256 × 256, slice thickness = 0.5 mm, slice gap of 0.1 mm, 34 slices, voxel size = 0.078 × 0.078 mm]. For the additional rationale of infant rat model brain scanning at PD14 in relation to *resting-state* functional MRI, please refer to our previous publication (33).

### 2.4. MRI data analysis

#### 2.4.1. Preprocessing

Raw MRI data was exported in dicom format and was transformed into Nifti files using the software dcm2nii. All images had their pixel dimensions scaled up in the Nifti header by a factor of 10 to avoid scale-dependent issues when using the FMRIB Software Library (FSL; http://www.fmrib.ox.ac.uk/fsl). T2-weighted images were first oriented using the software Freeview (v. 6.0; http://surfer.nmr.mgh.harvard.edu/fswiki/FreeSurferWiki). All images were rotated in the axial and coronal planes to achieve a uniform, upright, horizontal alignment to aid in subsequent manual segmentation.

#### 2.4.2. Segmentation

**Figure 2:**
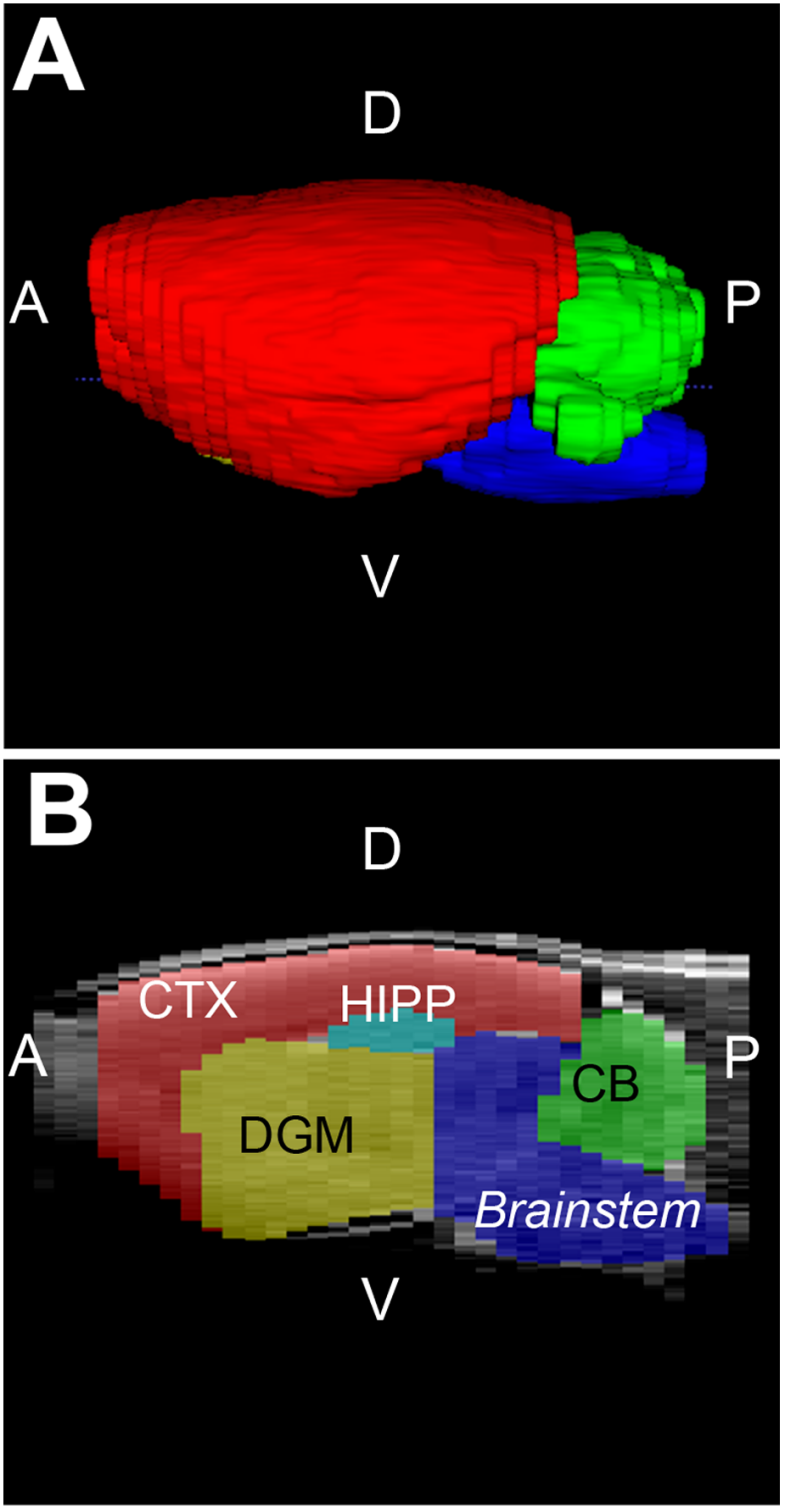
**Brain regions of interest.** Figure illustrates representative infant rat brain with selected brain region masks in 3D view (**A**) and in sagittal section (**B**). Brain regions analyzed included brainstem (blue), cerebellum (CB; green) and forebrain. The latter was further sub-divided into cortex (CTX; red), deep gray matter (DGM; yellow), and hippocampus (HIPP; teal). A, anterior; D, dorsal (posterior); *P*, posterior (caudal), V, ventral (inferior).

**Figure 3:**
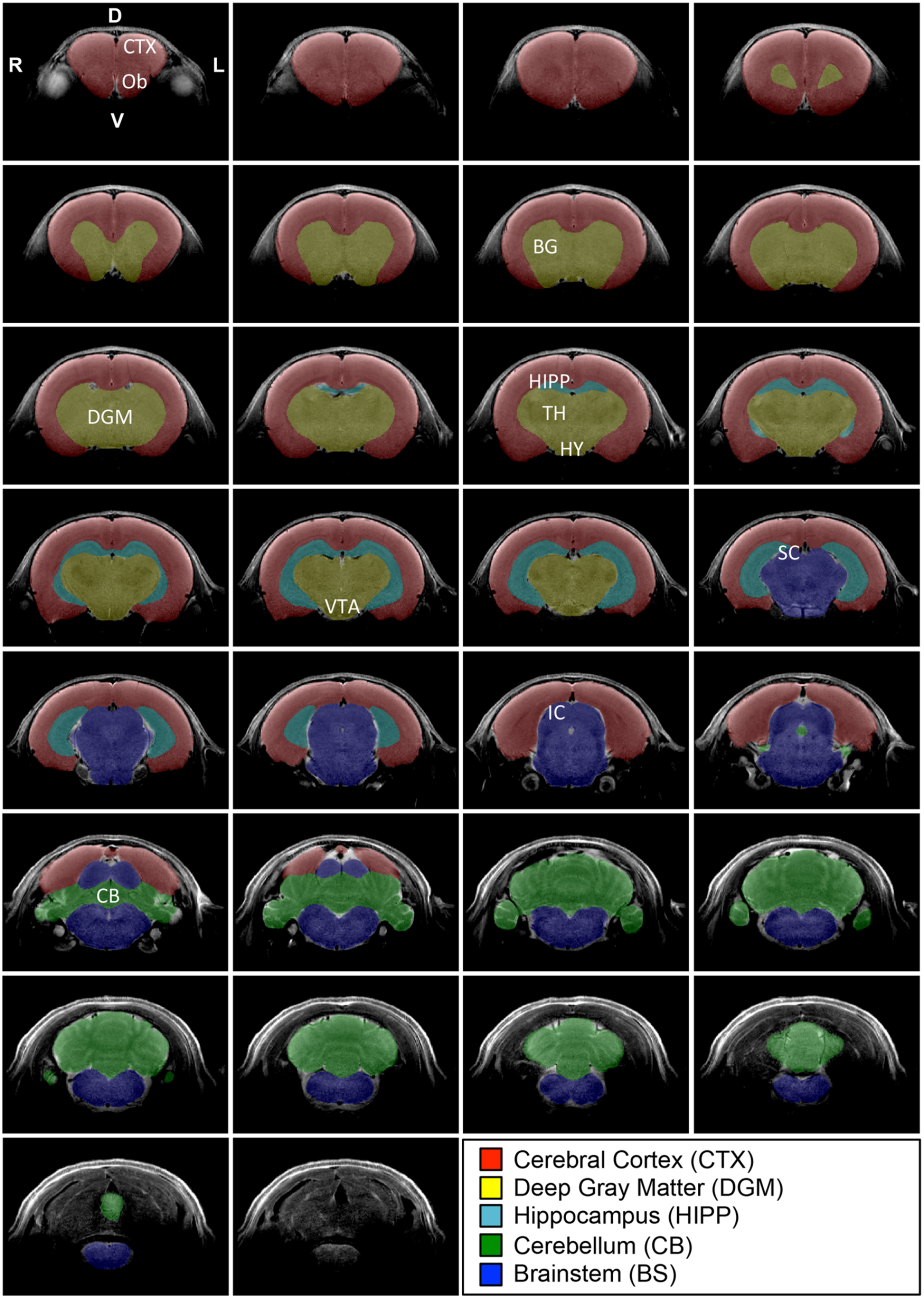
**Regional brain segmentation.** Figure systematically outlines representative T2-weighted coronal sections with its 5 regional brain segmentations in an infant rat at 2 weeks of age. Coronal brain sections are organized from the most rostral (top left) to the most caudal (bottom right) ends. The most anterior section included the last section-containing cortex and excluded those that solely contained olfactory bulbs (Ob; not shown). Similarly, the most caudal section for the masking included the last tissue from the cerebellum (CB) and excluded more caudal sections solely with medulla (bottom coronal section without any masks). We used ITK-SNAP (v.3.6.0; www.itksnap.org) for regional brain segmentation that included cortex (CTX; red), deep gray matter (DGM; yellow), hippocampus (HIPP; teal), cerebellum (green), and brainstem (BS; blue). DGM included all deep structures of the forebrain (e.g., basal ganglia (BG), thalamus (TH), hypothalamus (HY), ventral tegmental area (VTA). D, dorsal (posterior); IC, inferior colliculus; P, posterior (caudal), SC, superior colliculus; V, ventral (inferior).

A single investigator with neuroanatomical expertise blindly performed data analysis, which was subsequently checked by a senior researcher. Although automatic adult rat brain extraction software is available (34–37), issues of reliably are important in the segmentation of the infant rat brain (38). This relates to the low image contrast as a result of poor myelination in the infant rat brain (39) which poses a challenge for automatic segmentation protocols (40). To ensure consistency in analysis, both infant and adult rat images were manually segmented using the structural MRI tool ITK-SNAP (v.3.6.0; www.itksnap.org). Figure 2 illustrates major brain regions that were segmented, including the brainstem, cerebellum, and forebrain. The forebrain was further subdivided into the cortex, hippocampus, and deep gray matter. The deep gray matter comprises the basal ganglia and thalamus, structures known to be affected by chronic morphine administration (41, 42). Figure 3 illustrates coronal sections of the infant rat brains that aided in consistent segmentation of the infant brain regions [see also *MRI Atlas of the Infant Rat Brain* (43)]. Anatomical delineations were done according to the *The Rat Brain* atlas by Paxinos and Watson (44). Criteria for manual segmentation of rat brain tissue were as follows: *the most caudal section* includes cerebellar tissue while *the most rostral section* includes cortical tissue (Figure 3). Coronal slices containing only the spinal cord and olfactory bulb tissue (the most caudal and rostral, respectively) were excluded from the brain regional masks. Extra-axial cerebrospinal fluid and large vasculature exterior to the brain, as well as small areas of the ventricular system, were excluded from segmented regions. Lastly, the trigeminal ganglia were included as part of the brainstem segmentation according to the stereotaxic rat brain atlas by Schwarz's group (45).

### 2.5. End-point analyses

#### 2.5.1. Body weight

We previously showed that repeated morphine treatment in infant rats is associated with smaller body weight in comparison to controls [Figure1 in (15)]. We also reported that by adulthood, both sexes overcame the weight difference, although male adult rats weighed more than female adult rats. We extend those findings in the current report to assess the impact of subsequent pharmacological treatment in adulthood.

#### 2.5.2. MRI data volumes

ITK-SNAP was used to obtain the absolute (cm^3^) regional brain volumes of both infant and adult rats. Total brain volume was calculated as the sum of all 5 regions analyzed. Volumes were not only reported as absolute volumes (cm^3^) but also as normalized values (% total brain volume) to correct for possible individual data variations (46). Normalizing also allows for a better understanding of how a brain region might respond differently than the rest of the brain under specific pharmacological conditions.

### 2.6. Statistical analysis

Due to the split-litter design, the analysis unit was based on the number of individuals per treatment group (28). As in previous behavioral studies (15), we did not find any significant differences between male and female infant rats (6 female and 5 male saline-treated pups, 8 female and 4 male morphine-treated pups), so data for each infant pharmacological treatment group were collapsed for clarity. In contrast, due to volumetric differences found between male and female adult rats within pharmacological groups, data for the two sexes were analyzed separately. We used either a two-tailed independent Student's *t*-test or a one-way analysis of variance (ANOVA) with Tukey's Honestly Significant Difference (HSD) post-hoc tests to account for multiple treatment comparisons, to minimize type 1 error; Therefore, to adjust for 3 planned comparisons between treatment conditions, we used a conservative *p*-value <0.05/3 (*p *< 0.017) as the criteria for statistical significance. All statistical analyses were done with VassarStats (http://vassarstats.net/), a website for statistical computation.

## 3. Results

### 3.1. Weight differences with sex

**Figure 4:**
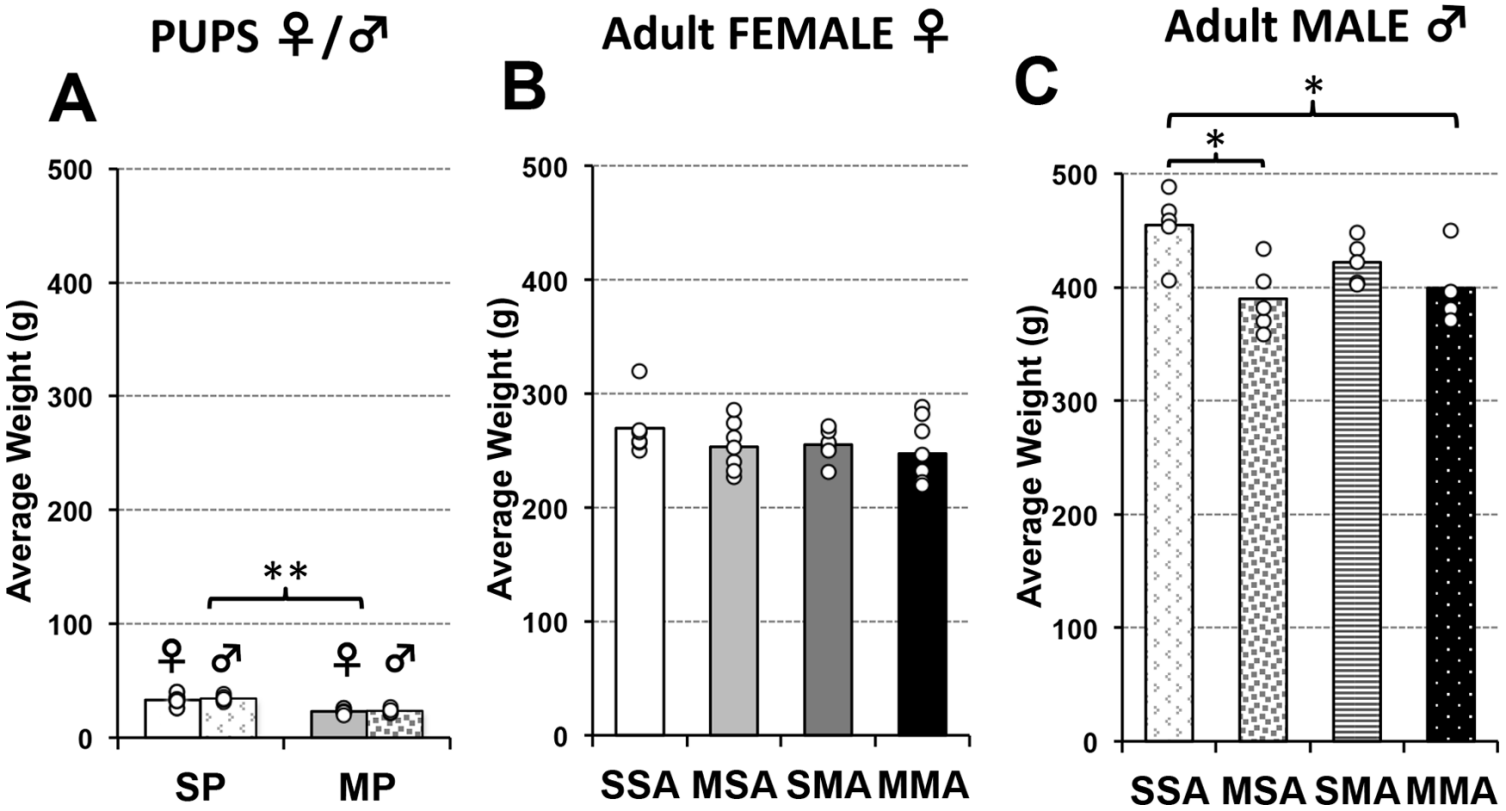
**Average body weight at brain MRI scan.** Graphs represent average body weight at brain MRI scan in grams (g) for infant (**A**) and adult rats (**B,C**). Infant rats pups were treated either with saline [saline pup (**SP**)] or morphine [morphine pup (**MP**)] twice daily for 2 weeks, which did not lead to weight differences in sex/group (SP (F vs. M), *F*(1,9) = 0.2; *p* = 0.66; MP (F vs. M), *F*(1,10) = 0.13; *p* = 0.73). Infant rats treated with morphine were smaller than those treated with saline [*F*(1,21) = 70.7, *p* < 0.001] without sex differences (**A**). Separate group of animals subsequently received additional 2 weeks of treatment in adulthood comprising total of 4 pharmacological groups (see also Figure 1): saline in infancy and saline in adulthood (SSA); morphine in infancy and saline in adulthood (MSA); saline in infancy and morphine in adulthood (SMA); and morphine in infancy and morphine in adulthood (MMA). Due to obvious sex differences in weight in adulthood, average weight of adult rats is separated by sex (**B,C**). Although trend in average body weight per pharmacological group was similar, group differences were found for male *F*(3,15) = 4.9, *p* = 0.015) but not female rats [*F*(3,23) = 1.1, *p* = 0.39]. ANOVA: *p < 0.05, **p < 0.01.

Similarly to our previous work (15, 25, 26), there were no significant body weight differences in infant rats across the sexes in either saline-treated [*t*(9) = 0.2, *p* = 0.665] or morphine-treated [*t*(10) = 0.13, *p* = 0.726] groups. Based on the lack of differences in body weight, data analysis of male and female infant rats was combined for simplicity of data presentation (Figure 4A). This contrasts with weight analysis in adult rats where females always had lower body weight compared to males irrespective of pharmacological treatment (Figures 4B,C; Statistics not shown). Therefore, all analyses for adult rats were done separately for males and females.

### 3.2. Weight differences with pharmacological treatment

Following two weeks of twice daily pharmacological treatment (Figure 1), morphine-treated infant rats had significantly smaller body mass (23.17 g ± 2.17) than saline-treated infant rats [33.82 g ± 5.37; *t*(21) = 70.74, *p* < 0.001]. In adult female rats, no weight differences were observed irrespective of infant or adult pharmacological treatment [*F*(3,23) = 1.06, *p* = 0.385, Figure 4B]. In contrast, we found significant differences in weight between pharmacological groups in adult male rats [*F*(3,15) = 4.88, *p* = 0.015, Figure 4C]. Specifically, male rats treated with morphine in infancy show lower body weight compared to saline control, regardless of treatment in adulthood (*p* < 0.05).

### 3.3. Prolonged morphine effects on total and regional brain volumes in an infant rat

**Figure 5:**
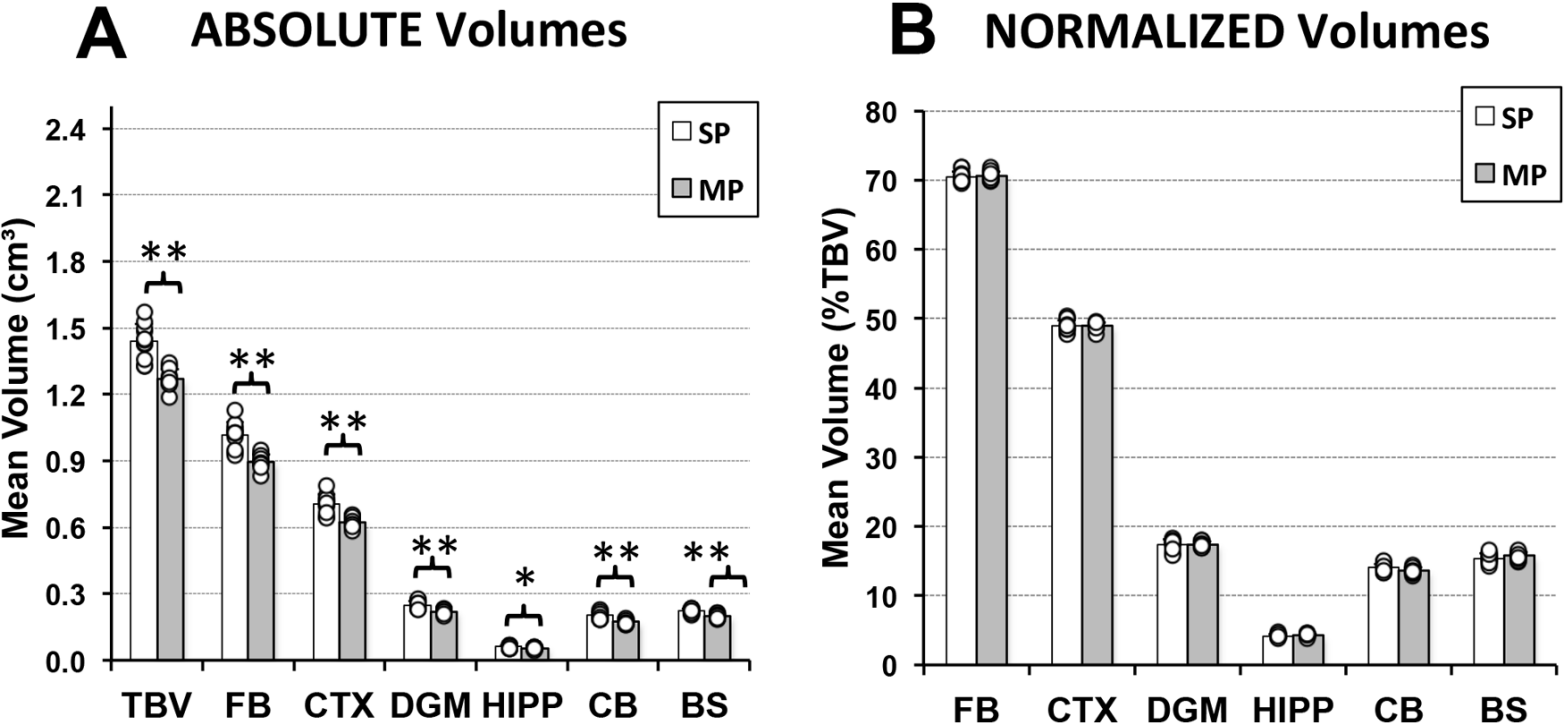
**Immediate effects of prolonged morphine treatment on total and regional brain volumes in an infant Rat.** Graphs show average *absolute* (cm^3^; **A**) and *normalized* brain volumes as a percent of total brain volume (%TBV; **B**) for the 2 groups of infant rats treated with saline [saline pup (SP); *n* = 11] or morphine [morphine pup (MP); *n* = 12] twice daily for 2 weeks. Absolute brain volumes of morphine-treated pups (MP) were smaller in comparison to saline-treated pups (SP) for total brain volume (TBV) and across all regions analyzed (**A**). No differences were observed in *normalized* brain volumes between groups (**B**). BS, brainstem; CB, cerebellum; CTX, cerebral cortex; DGM, deep gray matter; FB, forebrain; HIPP, hippocampus; ANOVA: *p < 0.05, **p < 0.01.

Since no sex-related brain volume differences were observed in infant rat groups, sex data was combined for clarity. As illustrated in Figure 5A, infant rats treated with morphine (*n* = 12) had consistently smaller total and regional brain volumes in comparison to controls (*n* = 11). Specifically, average total brain volume (cm^3^ ± SD) was significantly smaller in the morphine-treated group (1.23 ± 0.045) in comparison to the saline group [1.44 ± 0.079; *t*(21) = 41.7, *p* < 0.001]. Similar findings of smaller absolute volumes were observed across all regions analyzed: forebrain [*t*(21) = 36.92, *p* < 0.001], cortex [*t*(21) = 33.33, *p* < 0.001], deep gray matter [*t*(21) = 25.4, *p* < 0.001], hippocampus [*t*(21) = 0.005], cerebellum [*t*(21) = 25.53, *p* < 0.001], and brainstem [*t*(21) = 30.13, *p* < 0.001]. Collectively, these findings implicate globally lower total and regional brain volumes following prolonged morphine treatment in infancy. Indeed, this conclusion is supported by a lack of differences in normalized regional brain volumes (as % of total brain volume) between the two treatment groups. Specifically, we report no differences in normalized regional brain volumes between the two infant pharmacological groups (Figure 5B): forebrain [*F*(21) = 0.17, *p* = 0.68], cortex [*F*(1, 21) = 0, *p* = 1.00], deep gray matter [*F*(1, 21) = 0.01, *p* = 0.92], hippocampus [*F*(1, 21) = 0.55, *p* = 0.47], cerebellum [*F*(1, 21) = 4.56, *p* = 0.05], and brainstem [*F*(1, 21) = 1.81, *p* = 0.19].

### 3.4. Long-term effects of prolonged morphine administration in infancy on total and regional brain volumes in adult rat

**Figure 6:**
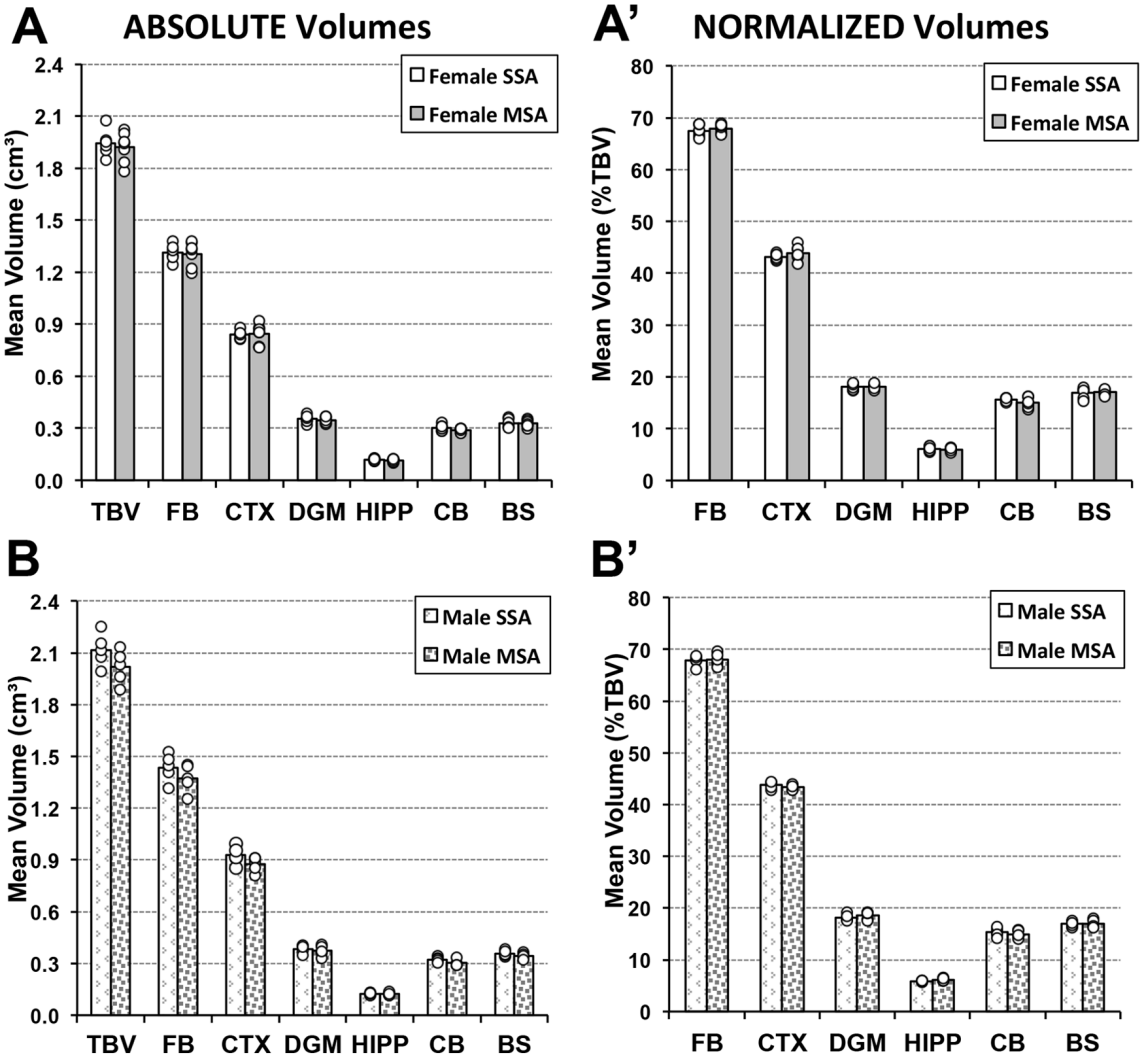
**Long-term effects of prolonged morphine treatment in infancy on adult Rat total and regional brain volumes.** Average absolute (cm^3^) and normalized brain volumes as a percent of total brain volume (%TBV) for female (**A** and **A**′, respectively) and male (**B** and **B**′, respectively) adult rats for the 2 groups of animals that underwent 2-week periods of treatment in infancy and adulthood: saline in infancy and saline in adulthood (SSA; *n* = 6 female; *n* = 5 male) and morphine in infancy and saline in adulthood (MSA; *n* = 7 female; *n* = 5 male). There were no significant differences in either absolute or normalized total or regional brain volumes between pharmacological groups for either sex using *t*-test. BS, brainstem; CB, cerebellum; CTX, cerebral cortex; DGM, deep gray matter; FB, forebrain; HIPP, hippocampus.

Due to previously established sex differences in weight of adult rats (Figure 4), we separated the brain volume analyses by sex. We compared two groups of adult animals who underwent either saline or morphine treatment in infancy followed by saline treatment in adulthood (SSA vs. MSA; Figure 1) to assess the long-term impact of morphine treatment in infancy. We report no differences in either absolute or normalized total and regional brain volumes in either female or male adult rat groups (Figure 6).

#### 3.4.1. Absolute volumes

There were no significant differences in total brain volumes of adult female rats treated with morphine in infancy (MSA *n* = 7; 1.92 cm^3^ ± 0.087) compared to those treated with saline [SSA *n* = 6; 1.95 cm^3^ ± 0.075; *t*(11) = 0.3, *p* = 0.59]. As further illustrated in Figure 6A, there were no differences in volumes of any of the brain regions analyzed: forebrain [*t*(11) = 0.07, *p* = 0.80], cortex [*t*(11) = 0.03, *p* = 0.87], deep gray matter [*t*(11) = 0.26, *p* = 0.62], hippocampus [*t*(11) = 0.68, *p* = 0.43], cerebellum [*t*(11) = 2.96, *p* = 0.11], and brainstem, [*t*(11) = 0.05, *p* = 0.83]. Similarly, male rats showed no difference in total brain volume between morphine- (MSA *n* = 5; 2.02 cm^3^ ± 0.097) and saline- (SSA *n* = 5; 2.12 cm^3^ ± 0.097) treated groups in infancy [*t*(8) = 2.66, *p* = 0.14]. Figure 6B further shows a lack of regional volume difference between SSA vs. MSA male groups: forebrain [*t*(8) = 1.68, *p* = 0.23], cortex [*t*(8) = 3.14, *p* = 0.11], deep gray matter [*t*(8) = 0.43, *p* = 0.53], hippocampus [*t*(8) = 0.13, *p* = 0.73], cerebellum [*t*(8) = 4.84, *p* = 0.059], and brainstem [*t*(8) = 2.0, *p* = 0.20].

#### 3.4.2. Normalized volumes

Morphine treatment in infancy had no impact on adult measures of normalized regional brain volumes for either sex. Female rats treated with morphine in infancy and saline in adulthood (MSA) did not have significantly different normalized regional brain volumes compared to those twice treated with saline (SSA), as shown in Figure 6A′: forebrain [*t*(11) = 0.79, *p* = 0.39], cortex [*t*(11) = 1.4, *p* = 0.26], deep gray matter [*t*(11) = 0.06, *p* = 0.81], hippocampus [*t*(11) = 0.39, *p* = 0.55], cerebellum [*t*(11) = 1.9, *p* = 0.20], or brainstem [*t*(11) = 0.04, *p* = 0.85]. Similarly, there was no significant differences in the normalized brain volumes measures for any of the regions analyzed in the male rat group either (Figure 6B′): forebrain [*t*(8) = 0.14, *p* = 0.72], cortex [*t*(8) = 1.14, *p* = 0.32], deep gray matter [*t*(8) = 0.61, *p* = 0.46], hippocampus [*t*(8) = 5.43, *p* = 0.05], cerebellum [*t*(8) = 0.39, *p* = 0.55], or brainstem [*t*(8) = 0.02, *p* = 0.89].

### 3.5. Effect of prolonged morphine administration in adulthood on total and regional brain volumes in the setting of previous morphine administration in infancy

**Figure 7:**
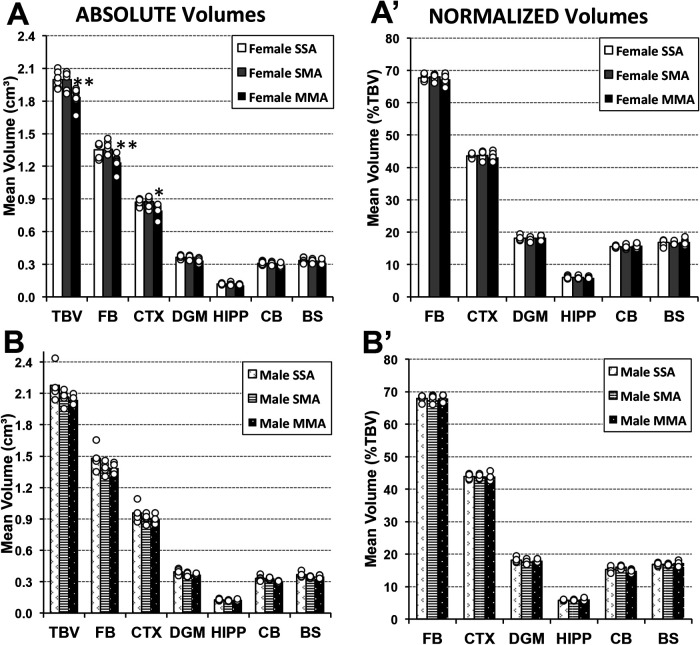
**Total and regional brain volumes following prolonged morphine administration in adult rats ± previous exposure to morphine in infancy.** Average absolute (cm^3^) and normalized brain volumes as a percent of total brain volume (%TBV) for female (**A** and **A**′, respectively) and male (**B** and **B**′, respectively) adult rats for the 3 groups of animals that underwent 2-week periods of treatment in infancy and adulthood: saline in infancy and saline in adulthood (SSA; *n* = 6 female; *n* = 5 male), saline in infancy and morphine in adulthood (SMA; *n* = 6 female; *n* = 5 male), and morphine in infancy and morphine in adulthood (MMA; *n* = 8 female; *n* = 4 male). There were no significant differences in average absolute total or regional brain volumes for either of sex following prolonged morphine administration only in adulthood (SSA vs. SMA). However, prolonged morphine administration in adulthood was associated with smaller total brain volume of female rats due to decreased absolute volumes of the forebrain and cortex. Cerebellum volume differences in males [*F*(2, 11) = 5.49, *p* = 0.022] are not considered significant due to conservative *p* value to account for multiple comparisons (significance *p* < 0.017). *Abbreviations*: BS, brainstem; CB, cerebellum; CTX, cerebral cortex; DGM, deep gray matter; FB, forebrain; HIPP, hippocampus; TBV, total brain volume. ANOVA: *p < 0.05, **p < 0.01.

The impact of morphine treatment in adulthood following morphine exposure in infancy was explored through the comparison of three groups: (1) morphine-treated adults who had undergone morphine treatment in infancy, (2) morphine-treated adults who received saline treatment in infancy, and (3) twice saline-treated control group (MMA, SMA, and SSA, respectively; Figure 1). Prolonged morphine administration in adulthood was associated with smaller total and regional brain volumes that vary by sex– but only in case of previous exposure to morphine in infancy (Figure 7).

#### 3.5.1. Absolute volumes

We report smaller total brain volumes in adult female rats twice treated with morphine [*F*(2, 17) = 9.06, *p* = 0.002] but not males [*F*(2, 11) = 5.49, *p* = 0.022] compared to the adult saline-treated groups, irrespective of the pharmacological treatment in infancy. Furthermore, prolonged morphine administration in adulthood was associated with smaller total brain volume of female rats due to decreased absolute volumes of the forebrain [*F*(2, 17) = 8.73, *p* = 0.003] and cortex [*F*(2, 17) = 6.83, *p* = 0.007]—but only in case of previous exposure to morphine in infancy (Figure 7A). Other regions showed no difference in absolute volumes between treatment groups: deep gray matter [*F*(2, 17) = 5.4, *p* = 0.015, group comparisons were not significant], hippocampus [*F*(2, 17) = 2.86, *p* = 0.085], cerebellum [*F*(2, 17) = 3.37, *p* = 0.059], and brainstem [*F*(2, 17) = 3.58, *p* = 0.05]. Although male rats showed consistently lower regional brain volumes following morphine treatment in adulthood, we failed to detect any group differences (Figure 7B): forebrain [*F*(2, 11) = 2.08, *p* = 0.17], cortex [*F*(2, 11) = 1.37, *p* = 0.29], deep gray matter [*F*(2, 11) = 3.13, *p* = 0.08], cerebellum [*F*(2, 11) = 5.49, *p* = 0.022], hippocampus [*F*(2, 11) = 1.42, *p* = 0.28], and brainstem [*F*(2, 11) = 1.58, *p* = 0.25]. Data for cerebellar volumes was not considered significant due to conservative *p*-value to account for multiple comparisons (significance *p* < 0.017).

#### 3.5.2. Normalized volumes

* *Based on described region-specific brain volume differences, we would expect group differences in normalized volumes correlated with the significant results outlined above. However, we observed no significant normalized volume differences for any of the regions for female rats between treatment groups: forebrain [*F*(2, 17) = 0.57, *p* = 0.58], cortex [*F*(2, 17) = 0.47, *p* = 0.63], deep gray matter [*F*(2, 17) = 0.09, *p* = 0.91], hippocampus [*F*(2, 17) = 0.31, *p* = 0.74], cerebellum [*F*(2, 17) = 0.81, *p* = 0.46], and brainstem [*F*(2, 17) = 0.28, *p* = 0.76] (Figure 7A′). Similarly, adult male rats displayed no differences in normalized volumes between treatment groups for any region: forebrain [*F*(2, 11) = 0.85, *p* = 0.453], cortex [*F*(2, 11) = 0.03, *p* = 0.971], deep gray matter [*F*(2, 11) = 0.72, *p* = 0.508], hippocampus [*F*(2, 11) = 1.58, *p* = 0.249], cerebellum [*F*(2, 11) = 3.02, *p* = 0.09], and brainstem [*F*(2, 11) = 0.84, *p* = 0.458] (Figure 7B′).

## 4. Discussion

We report smaller body weight and globally lower absolute brain volumes in infant rats following prolonged morphine treatment. These differences in both body weight and brain volume resolved in adulthood. However, upon initial exposure or re-exposure to morphine in adulthood, a decrease in the volume of select brain regions was observed that differed by sex. Lack of normalized volume differences in adult female rats could, in part be explained by small data differences between groups and relatively gross regional brain analysis.

### 4.1. Study limitations

This study should be interpreted with a few limitations in mind. The morphine-treated infant rat group included twice as many female pups (*n* = 8) as males (*n* = 4), which similarly translated to twice as many twice morphine-treated adult female rats (*n* = 8) as males (*n* = 4). Future studies with greater power are needed to confirm our results. Furthermore, our method of manual tissue segmentation as well as the more challenging anatomical borders in infant rats prevented the delineation of smaller, specific structures of interest previously shown to exhibit structural changes in response to morphine administration in infancy, such as the amygdala (26). With higher resolution imaging, and the advent of more powerful automated segmentation techniques, future studies of structural differences are needed to evaluate vulnerability of specific brain areas beyond gross brain regional divisions.

### 4.2. Body mass with age, sex, and pharmacological treatment

As previously reported by our group (15, 26), we observed slower body weight gain during prolonged morphine administration in infancy. The lower body weight of infant rats treated with morphine could, in-part be attributed to the fact that morphine-treated animals were asleep longer following injections. Due to the split-litter design, saline-treated pups were therefore conferred a nursing advantage over their morphine-treated siblings within the same litter. Such restrictions in diet and feeding schedules have previously been shown to induce rapid weight loss (47). Interestingly, recent study by O'Meara et al. (48) reported that morphine treated juvenile rats treated twice daily for 7 days (PD18–24) gained less weight than those treated with repeated benzodiazepine (viz. midazolam) or saline control, suggesting opioid behavioral effects on appetite and/or resource acquisition. Future studies could employ a different method of assigning pharmacological groups to determine the degree to which nursing advantage affects body weight. In addition, future studies should consider comparison of pharmacological effect of morphine to other sedating agents (e.g., midazolam, dexmedetomidine) in this early neurodevelopmental period (PD1–14) to better assess disrupted rodent diurnal patterns and reduced nighttime eating with associated weight loss—as was previously described in rats (49) and other species (50, 51).

Furthermore, we previously reported no difference in percent body mass gain during the morphine-weaning period between morphine- and saline-treated rats [see Figure 1B in (15)]. These findings indicate a recovery of body mass growth following the cessation of morphine dosing that may translate to the compensatory growth we observe before adulthood. Our novel results show weight differences in adulthood only for male rats following morphine exposure irrespective of the time-period of administration (Figure 4). Presented sex differences of prolonged morphine administration on body weight warrant future studies.

### 4.3. Immediate effect of prolonged morphine administration in infancy

Our data showed globally smaller brain volumes in infant rats that received prolonged morphine administration compared to saline-treated pups. These findings are in line with previous studies that (i) implicate decreased somatic development in morphine-treated rat pups as measured by body weight and brain weight and size (52, 53), as well as (ii) reported region-specific apoptosis in infant rats treated with morphine (26). We expanded on these studies by analyzing gross brain regions by MRI, and we report a uniform rather than localized reduction in brain volume in morphine-treated rat pups. Though all brain regions had lower absolute volumes in morphine-treated pups, only the normalized cerebellum volumes were close to significantly smaller (*p* = 0.05), indicating that the developing cerebellum may have an increased vulnerability to morphine. One possible explanation comes from a study by Zwicker, who found smaller cerebellum volumes in morphine-treated preterm infants (54). The authors concluded that morphine's effect on the cerebellum is likely mediated by the death of Purkinje cells, as morphine localizes to the nerve terminals surrounding these cells (55) and that this effect may be limited in rodents to the first few days of life (56). Certainly, future studies should explore changes in opioid receptors throughout the brain (57, 58) following prolonged morphine exposure as described in used infant rat model. Such inquiry would implicate pre/post-synaptic (mal)adaptations driven by opioid receptors that could potentially be linked to increased cortical apoptosis demonstrated previously (26).

### 4.4. Long-term effects of prolonged morphine administration in infancy

Adult rats previously treated with morphine for a prolonged time in infancy did not show differences in total or regional brain volume implicating neuroplasticity following prolonged morphine exposure during early brain development. Using the same rodent model, our group previously reported subtle long-term behavioral effects in adult rats at PD55–56 following prolonged morphine administration in infancy (15). Specifically, we reported selective long-term neurobehavioral differences in thermal but not mechanical sensory processing. Importantly, our results suggested a lack of long-term alterations on drug reward/reinforcement behavior [*via* locomotor testing (59, 60)], affective processing [using swim test (61)], and short-term memory [*via* novel-object recognition evaluation (62)]. Similarly, reduced nociceptive thresholds to thermal and mechanical testing (13), or formalin injections (14) at PD60 have been reported following daily administration of morphine (5 mg/kg from PD7–14). Future studies are needed to address the potential neuroplastic alterations at the molecular, cellular, and/or brain networks level using *resting-state* functional MRI to explore possible, subtle differences of thalamo-cortical pathways responsible for long-term morphine effects on sensory processing.

### 4.5. Prolonged morphine administration in adulthood in the setting of previous exposure in infancy

This study reveals decreased total and regional brain volumes in rats treated with morphine in adulthood, regardless of morphine treatment in infancy, that differ based on sex. It has been previously established that morphine treatment in infancy can have lasting behavioral effects that differ by sex, specifically with increased analgesia in adult females and decreased analgesia in males (63). This may implicate a higher degree of potency in adult female rats than males, which may be hormonal as suggested by gonadectomy studies (64, 65). Our morphological data provides further evidence to the sex based morphological differences in adult rats following prolonged morphine administration. Future studies are needed to address underlying cellular integrity (e.g., neuronal count, apoptosis) and possibly network level analysis using functional brain MRI. Previous studies have also described decreased morphine potency in adulthood, resulting from a reduced density of mu-opioid receptors after PD21 (66). Previous literature also reported reduced effectiveness of high doses of morphine in adult rats (67, 68), which supports our findings that morphine-treated pups had globally smaller brain volumes while morphine-treated adults only had regionally smaller brain volumes. Longitudinal studies performed at the cellular level are needed to determine if the mechanisms of morphine-induced structural changes in infancy may be impacted on subsequent exposure in adulthood.

### 4.6. Translational aspects: animal-human correlation

The association of rat and human developmental stages has been previously described and extensively discussed in the literature. In fact, the infant rat model at PD7 and PD14 has been extensively used in relation to early (premature, neonatal, and infant) and childhood development in humans, respectively (69, 70). Such difficult species developmental associations depend on several endpoints, such as number of brain cells, degree of myelination, brain growth rate, synaptogenesis, and measures related to more contemporary neuroinformatics (71, 72). In rodents, this critical period of neuronal differentiation and synaptic development is limited to a time window up to a 4th postnatal week [PDs 1–28; (73–75)]. In humans, the described brain growth spurt characterized with synaptogenesis and accompanied by dendritic and axonal growth, as well as myelination of the subcortical white matter, extends from the last trimester of pregnancy up to the first few years of postnatal life (76).

### 4.7. Clinical relevance

#### 4.7.1. Brain size

In humans, low birth weight has been correlated with morphological changes throughout life including lower total brain volume and smaller cortical surface area, even in children born full term (77). These findings indicate a correlation between weight and brain volume that may help explain our findings of smaller absolute brain volumes in morphine-treated rat pups. However, adolescents with low birth weights who later attained a normal body weight showed no significant difference in brain volume measures compared to control participants (78, 79), which aligns with our lack of observed differences in adult rat brain volumes. Other studies have found significant differences in regional brain volumes of school-aged children independent of birth weight following **prenatal** opioid exposure (80), which suggests a weight-independent mechanism of lower brain volumes because of opioid treatment. Our results build on these findings in a postnatal model, suggesting that morphine administration in infancy does not result in permanently smaller brain regions, but that such treatment may impose a vulnerability to reduced regional brain volumes when treated again with morphine in adulthood.

#### 4.7.2. Long-term effects of opioid exposure in infancy

While many studies probe the effects of **prenatal** opioid exposure (81, 82), those that focus on postnatal opioid exposure are scarce (81, 82). Studies of school-aged children who were **prenatally** exposed to opioids reported lower volumes of specific brain regions, including the basal ganglia, thalamus, and cerebellum (80, 83, 84). Furthermore, compared executive function and attention have been reported in human children exposed to morphine **prenatally** (85–87), but confounding factors make it difficult to isolate morphine treatment as a risk factor. Indeed, postnatal opioid exposure that is prolonged and associated with developmental of tolerance and iatrogenic dependence to drugs of sedation (viz. opioids and benzodiazepines) occurs primarily in the perioperative settings (88) that implicates more than pharmacological impact on the brain in the context of other confounders (e.g., gestational age at birth, underlying disease severity, pain in the context of surgery, cumulative anesthesia exposure, etc.).

## 5. Conclusion

Considering opioids are still largely considered the “gold standard” of pain management for infants and children, it is important to elucidate the immediate and long-term neurodevelopmental impact of prolonged administration of opioids in developing brain. Our study shows for the first-time short- and long-term pharmacological effects of prolonged morphine administration in an infant rat model on the effects on the total and regional brain volumes in adulthood and its vulnerability to subsequent morphine administration.

## Data Availability

The raw data supporting the conclusions of this article will be made available by the authors, without undue reservation.
